# Biogas

**DOI:** 10.1111/1751-7915.12854

**Published:** 2017-09-14

**Authors:** Caroline M. Plugge

**Affiliations:** ^1^ Laboratory of Microbiology Wageningen University and Research Stippeneng 4 6708 WE Wageningen The Netherlands; ^2^ Wetsus, European Centre of Excellence for Sustainable Water Technology Oostergoweg 9 8911 MA Leeuwarden The Netherlands

## Abstract

Biogas production represents a fascinating process for the recovery of nutrients and renewable energy from various organic waste streams. The process is of interest for the production of value‐added chemicals by mixed cultures and can also be applied in combined bioenergy production systems. Strategies and opportunities for optimization of biogas quality and quantity are presented.

Biogas typically consists of methane (50%–75%) and carbon dioxide (25%–50%), minor amounts of other gases and water vapour (http://www.biogas-renewable-energy.info). Biogas is produced from complex organic material that is decomposed by microorganisms in the anaerobic digestion (AD) process. This ability has already been utilized for centuries in man‐made systems (bioreactors) for production of energy (Bond and Templeton, 2011). Biogas is a valuable energy source yielding 5.5–7 kWh m^−3^, and the energy content is directly linked with the methane content. Its role in the scenario of the future sustainable energy supply is both distinct and flexible, as it can be used as renewable source of electricity and heat as and when needed, and can be stored. It will help to reduce the use of fossil fuels and thus reduce CO_2_ emissions. Although estimated values vary significantly between studies, the potential to produce biogas from waste is enormous (http://european-biogas.eu/biogas/; IEA 2015a,b).

Determination of practical and theoretical biogas potential is very important for design for optimal process design, configuration and effective evaluation of the economic feasibility (Weiland, 2010). A wide variety of process applications for biomethanation of wastewaters, slurries and solid waste are well established. These utilize different reactor types (fully mixed, plug flow, upflow anaerobic sludge bed, etc.) and process conditions (retention time, loading rate, temperature, etc.) to maximize the energy recovery from waste and as well as decrease retention time and enhance process stability. Most often applied are wet digester systems using vertical stirred tank digester with different stirrer types dependent on the origin of the feedstock (Weiland, 2010; Schnurer, 2016).

To optimize the anaerobic digestion process and steer it in the desired direction, it is important to have in‐depth knowledge and understanding of the anaerobic microbiome, including metabolic capacities of the microorganisms, the degree of functional redundancy within the community as well as the mechanisms for interspecies interactions. Different physiological groups of microorganisms are involved as follows: hydrolytic bacteria, fermenting bacteria, organic acid‐oxidizing bacteria and methanogenic archaea, and these microorganisms degrade organic matter via cascades of biochemical conversions ultimately to biogas (Weiland, 2010). Syntrophic relationships between hydrogen producers (acetogens) and hydrogen scavengers (homoacetogens, hydrogenotrophic methanogens) are critical to the process (Plugge *et al*., 2010; Carballa *et al*., 2015).

Hydrolysis is the first step in AD and often considered as the rate‐limiting step in conversion of waste such as lignocellulosic biomass, primary sludge, industrial wastes and manure (Vavilin *et al*., 2008; Ma *et al*., 2013). Although extensive research has been performed to improve the understanding of the anaerobic digestion process, research on anaerobic hydrolysis and its microbiology is still poorly understood (Azman *et al*., 2015). As the biogas yield is depending on the extent of hydrolysis, research on the improvement of the hydrolysis step is required to enhance the overall AD process.

Start‐up period of the anaerobic digesters is crucial for stable and efficient biogas production (Escudié *et al*., 2011; Kim *et al*., 2013; Goberna *et al*., 2015). Start‐up is the required time in which a dedicated microbial community for AD of a specific waste stream can grow, develop and become stable and redundant. AD without a start‐up period may lead to inefficient organic matter conversion, consequently to inefficient biogas production, extended acclimation time to the selected compounds and unexpected process failures during the reactor operation (Griffin *et al*., 1998; Liu *et al*., 2002; Escudié *et al*., 2011). Many strategies have been reported to start up anaerobic bioreactors, focussing on the evaluation of selecting appropriate seed sludge, organic loading rates, inoculum/substrate ratio, temperature and/or the type of reactor. All lead to avoid accumulation of intermediate products such as volatile fatty acids which inhibit methanogenesis and limit biogas yield during the reactor operation. During start‐up, next‐generation sequencing (NGS) is an indispensable tool to screen the complex microbial community dynamics. Frequent utilization of NGS during start‐up period aids to monitor establishment of microbial communities within the bioreactors and helps to understand if all degradation pathways leading to biogas production are already established in the microbiome (Appels *et al*., 2011). Therefore, these methods should be used in parallel with the physico‐chemical monitoring during any start‐up of anaerobic bioreactors.

The biogas produced in AD does not meet the requirements for injection in existing gas distribution systems or use in other applications (Wellinger and Lindberg, 2001). For all applications, it should be considered that the pressure of the produced gas must exceed the pressure requirement of the applications or the local grid. In the concept of autogenerative high‐pressure digestion (AHPD), active methanogenic biomass builds up pressure inside the bioreactor (Lindeboom *et al*., 2011). As CO_2_ has a higher solubility than CH_4_, at higher pressures, it will fractionate more to the liquid phase, resulting in AHPD biogas with a high CH_4_ content (~90%–95% and 0.3–9 MPa). Ideally, high‐quality biogas can be directly used for electricity and heat generation, or injected in a local natural gas distribution net (Lindeboom *et al*., 2011). It is intriguing whether a relation exists between microbial communities enriched in high‐pressure AD today and those involved in the formation of natural gas fields that have very high pressures (e.g. 35 MPa for the Slochteren gas fields in Netherlands). Methanogens have been isolated from and detected in high‐pressure deep subsurface gas and oil reservoirs (Wu and Lai, 2011; Mayumi *et al*., 2013). From this perspective, understanding the microbial pathways and population dynamics in AHPD is fascinating and relevant not only from the biotechnological point of view, but also by offering potential insight into the origin of biogenic natural gas and the consequences of carbon capture in subsurface reservoirs (Mayumi *et al*., 2013).The major part of fossil natural gas has been produced by microbial degradation of organic matter, but the microbial pathways resulting in the formation of pressurized gas fields remain unknown. The effect of elevated *p*CO_2_ on Gibbs free energy, microbial community composition and substrate utilization kinetics in AHPD revealed a generic role of the microbiome in biogas formation at elevated (up to 2 MPa) pressure (Lindeboom *et al*., 2016). The propionate conversion rate and subsequent methane production rate were inhibited by up to 90% by the accumulating *p*CO2 up to 0.5 MPa in the pressure reactor, and this toxicity was reversible. This opens opportunities for steering carboxylate production using the reversible CO_2_ toxicity in mixed‐culture microbial electrosynthesis and fermentation. To further develop the AHPD process, it is essential that depending on the type and concentration of the substrate and the desired biogas quality, a suitable operational pressure is chosen. New microbial methods and models for monitoring the efficiency and stability of the process need to be further developed to steer and manage AHPD towards higher efficiency and for controlled production.

The decomposition of food waste in landfills accounts for 23% of all methane emissions in the United States (US EPA, 2010). In a landfill, food waste decomposes rapidly and releases biogas in a totally uncontrolled manner, where it contributes to global warming as it is at least 25 times more powerful than CO_2_ (Myhre *et al*., 2013). Here, combining hydrothermal liquefaction and anaerobic digestion may yield a higher energetic return by converting the food waste (or other feed stocks) into oil and biogas and further recovery of resources (digestate). Hydrothermal liquefaction converts waste into oil and a carbon‐rich aqueous phase that can be converted to biogas via AD (Gerber Van Doren *et al*., 2017; Posmanik *et al*., 2017). A mixture of polysaccharides, proteins and lipids, representing food waste, underwent hydrothermal processing at temperatures ranging from 200 to 350°C. The anaerobic biodegradability of the hydrothermal aqueous phase was examined through conducting biochemical methane potential assays. It was shown that the anaerobic biodegradability of the hydrothermal aqueous phase was lower when the temperature of hydrothermal processing increased and the chemical composition of the hydrothermal aqueous phase affected the anaerobic biodegradability. However, no inhibition of biodegradation was observed for most samples (Gerber Van Doren *et al*., 2017; Posmanik *et al*., 2017). A proper analysis of all carbon flows is essential as a design tool for process integration as it may provide a good estimation for resource recovery based on the final product that is targeted (oil or biomethane), the system thermal capabilities and feedstock composition. Moreover, it makes optimal use of the power of microbes in the production of biogas. Knowledge of digester technologies and process microbiology has grown rapidly in recent years, and has reached a point where the process is set up and managed under controlled conditions. However, AD digestion and biogas production at more extreme conditions such as high or low pH, high salinity or with recalcitrant biomass still are understudied.

Another important factor for further enlargement of biogas production is the development of small‐scale, cheap and efficient technologies for use at farm scale to reach the full potential and to access the high biogas potential in the agricultural sector. An adjustment of AHPD technology to required biogas quality and available resources of rural communities makes this technology more widely applicable. Small‐scale solutions are of importance in non‐industrialized and developing countries, where AD can be of great importance for a whole country, as well as families.

## Conflict of interest

None declared.
